# Racial and Ethnic Disparities in Peri-and Post-operative Cardiac Surgery

**DOI:** 10.1007/s12170-024-00739-4

**Published:** 2024-07-29

**Authors:** Shane S. Scott, Doug A. Gouchoe, Lovette Azap, Matthew C. Henn, Kukbin Choi, Nahush A. Mokadam, Bryan A. Whitson, Timothy M. Pawlik, Asvin M. Ganapathi

**Affiliations:** 1 Medical Scientist Training Program, Biomedical Sciences Graduate Program, The Ohio State University, Columbus, OH USA; 2 Division of Cardiac Surgery, Department of Surgery, The Ohio State University Wexner Medical Center, N-809 Doan Hall, 410 W. 10th Ave, Columbus, OH 43210 USA; 3 COPPER Laboratory, Department of Surgery, The Ohio State University Wexner Medical Center, Columbus, OH 43210 USA; 4 Department of Surgery, The Ohio State University Wexner Medical Center, Columbus, OH USA

**Keywords:** Cardiac surgery, Transplantation, Race, Disparities, Health equity, Socioeconomic status

## Abstract

**Purpose of Review:**

Despite efforts to curtail its impact on medical care, race remains a powerful risk factor for morbidity and mortality following cardiac surgery. While patients from racial and ethnic minority groups are underrepresented in cardiac surgery, they experience a disproportionally elevated number of adverse outcomes following various cardiac surgical procedures. This review provides a summary of existing literature highlighting disparities in coronary artery bypass surgery, valvular surgery, cardiac transplantation, and mechanical circulatory support.

**Recent Findings:**

Unfortunately, specific causes of these disparities can be difficult to identify, even in large, multicenter studies, due to the complex relationship between race and post-operative outcomes. Current data suggest that these racial/ethnic disparities can be attributed to a combination of patient, socioeconomic, and hospital setting characteristics.

**Summary:**

Proposed solutions to combat the mechanisms underlying the observed disparate outcomes require deployment of a multidisciplinary team of cardiologists, anesthesiologists, cardiac surgeons, and experts in health care equity and medical ethics. Successful identification of at-risk populations and the implementation of preventive measures are necessary first steps towards dismantling racial/ethnic differences in cardiac surgery outcomes.

## Introduction

**Fig. 1:**
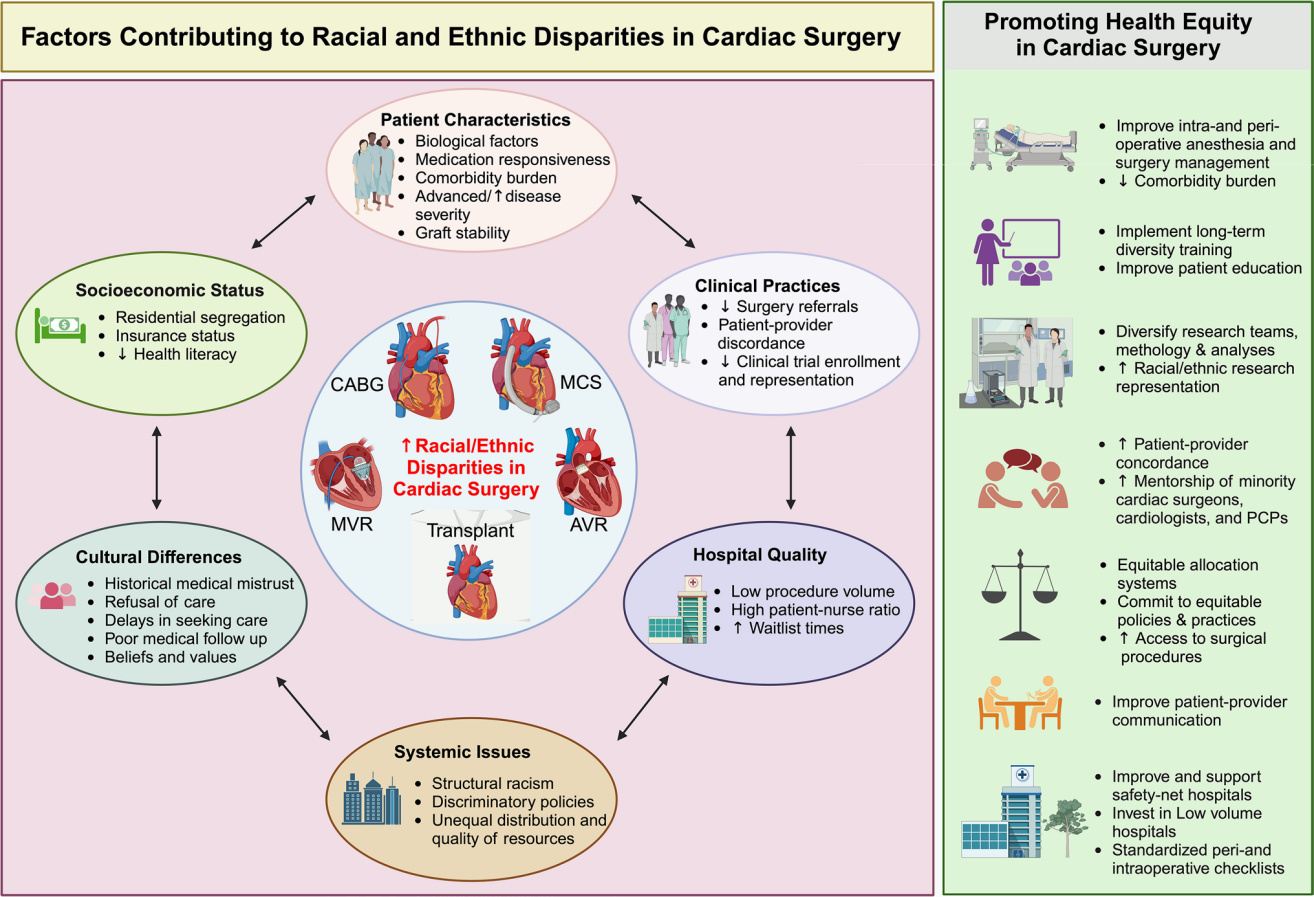
Race and Ethnicity in Cardiac Surgery. Several factors including patient characteristics, clinical practice, systemic issues, hospital quality, socioeconomic status, and cultural differences have been shown to influence racial/ethnic disparities in cardiac surgery. Despite these barriers, proposed solutions require deliberate actions from culturally sensitive and diverse medical care teams. Created with BioRender.com. AVR = aortic valve replacement; CABG = coronary artery bypass graft; MCS = mechanical circulatory support; MVR = mitral valve replacement/repair; PCP = primary care physicians

Despite being a poorly defined social construct, race has emerged as a powerful risk factor in peri-and post-operative morbidity and mortality in cardiac surgery [1, 2]. While advancements in cardiac surgery have reduced morbidity and mortality, improvement in care has not been uniform across racial/ethnic groups [3, 4]. Though racial and ethnic minority groups are underrepresented in clinical trials and research studies, Black and Hispanic patients in particular have worse outcomes compared with White patients following cardiac surgery [3, 5]. These race-based differences have been attributed to a combination of patient characteristics [1, 6, 7, 8], socioeconomic status (SES) [7, 8, 9–11], clinical practices, cultural differences, hospital quality [7, 8] and systemic issues (Fig. 1). Recent studies also suggest that disparities in cardiac rehabilitation may offer a potential explanation for differing outcomes due to underutilization of this life-saving therapy by racial/ethnic minority groups [12, 13]. However, even after accounting for these differences, a significant portion of race-based differences cannot be explained, thus warranting further investigation towards reducing racial/ethnic disparities in healthcare. The aim of this review is to highlight known racial/ethnic disparities in several cardiac surgical procedures including coronary artery bypass surgery (CABG), valvular surgery, cardiac transplantation, and mechanical circulatory support (MCS), and to describe their associations with peri- and post-operative morbidity and mortality. Of note, all referenced comparisons are to white individuals unless stated otherwise. Patient and hospital characteristics that impact post-operative outcomes among these patients are also documented. In addition, we explore factors that contribute to increased prevalence of disparities and propose potential solutions to help guide development of sustainable and equitable solutions.

## Coronary Artery Bypass Graft (CABG)

While CABG remains one of the most effective treatments for coronary artery disease, several studies have demonstrated that racial background can significantly impact peri- and post-operative mortality in patients (Table 1) [7, 8, 14], with early mortality risk being 1.5 times higher for Black patients [15, 16]. A previous study using the Society of Thoracic Surgeons (STS) National Database reported that Black race is an independent predictor of operative mortality during CABG [14]. Unfortunately, these differences have persisted over the past few decades, despite improvements in operative techniques and adoption of more equitable practices. Additional studies using the Medicare database have noted that minority patients had a 33% higher risk-adjusted mortality rate after CABG [7]. Amongst Veteran Affair’s patients, individuals who identify as Black have even had higher mortality among low-risk patients, which was attributed to increased post-operative complications such as renal failure, respiratory complications, and bleeding [17]. A rigorous eighteen-year study by Becker and colleagues, with over five million patients, demonstrated that while in-hospital CABG mortality dramatically decreased between 1988 and 2015, significant gaps persisted between Black males and other minority groups [3]. Black male CABG patients had a 35.1% higher risk of in-hospital mortality. In contrast, after controlling for patient and hospital factors, Hispanic and Asian patients had significantly lower risks of in-hospital mortality. These data are supported by a recent meta-analysis that reported an increased risk of in-hospital mortality for Black patients [4], and a more recent STS study that demonstrated Black patients had higher in-hospital mortality (2.76% vs 2.19%) [18]. These differences persisted following discharge as well, from 3-months up to 10 years post-CABG [3, 15, 19]. Amongst Medicare patients, Black patients had higher 3-month and 1-year mortality [16]. Gray et al. further demonstrated reduced survival among Black patients at 1- and 5-years post CABG, which persisted following adjustments for pre-operative factors [20]. Supporting this finding, Cooper et al. noted that 3-, 5-, and 10-year survival for Black patients was lower [19].

**Table 1:** Racial and ethnic disparities in coronary artery bypass grafting

Author, Year	Study Design	Era	Sample Size	Population and % Ethnic Breakdown	Outcomes
Rumsfeld et al. [17]	Retrospective multicenter cohort study of patients who underwent CABG surgery at any one of the 43 VHA cardiac surgery centers	1995–2001	N = 33,428	White (87.7%), Black (7.7%), and Hispanic (4.6%),	Adjusted mortality risk in Black patients were similar, while Hispanic patients had lower risk compared to Whites. Among patients with low surgical risk, Black patients had higher mortality and were more likely to experience postoperative renal failure, respiratory complications, and bleeding
Cooper et al. [19]	STS Adult Cardiac Database at Emory for patients undergoing CABG (on or off-pump) records	1997–2007	N = 12, 874	White (84.2%), Black (15.8%)	Short- and long-term outcomes (3, 5, 10 years) were significantly worse in Black than in White patients undergoing CABG. On pump CABG did not narrow the disparity in outcomes between Blacks and Whites
Rangrass et al. [7]	Multicenter study using the National Medicare database with patients undergoing CABG at US hospitals	2007–2008	N = 173, 925	White (91.4%), Non-white (8.6%)	Non-white patients had higher risk-adjusted in-hospital mortality rates after CABG surgery than White patients. Differences in hospital quality explained 35% of the observed disparity in mortality rates, while 53% was explained by SES and hospital quality combined
Becker et al. [24]	Health Care Utilization Project (HCUP) with CABG patients	1998–2002	N = 1,192,540	White (87.3%), Black (5.4%), Hispanic (5.7%), and Asian-American/Pacific-Islander (1.7%)	Male and female Black CABG patients, had significantly worse in-hospital mortality rates than other races/ethnicities after controlling for patient and SES factors
Mehta et al. [8]	Society of Thoracic Surgery Database	2010–2011	N = 148 059	White (92.1%), Black (7.9%)	Following adjustment post CABG mortality and major morbidity rates were higher for Black individuals. Surgeon, hospital, and care processes in addition to patient and SES factors were included in the adjusted analysis
Becker et al. [3]	NIS data with over 5 million CABG patients	1998–2015	N = 5,032,985	White (63.4%), Black (4.8%), Hispanic (5.0), Asian (1.6), Other known race/ethnicity(2.9%), Unknown race/ethnicities (22.4%)	Black males had worse in-hospital CABG mortality than White male patients, while Hispanic, and Asian-American had significantly lower in-hospital CABG mortality rates
Konety et al. [16]	Retrospective cohort study of Medicare beneficiaries 65 and older undergoing CABG	1997–2000	N = 591,139	White (95.9%), Black (4.1%)	Black patients were more likely to undergo CABG at hospitals with the highest mortality and lowest volume quintile and had increased unadjusted and adjusted mortality
Benedetto et al. [4]*	A meta-analysis study composed of 28 CABG outcome studies	Inception-2017	N/A	Black, Hispanic, Asian, and White	Adjusted in-hospital mortality was greater for Black patients but similar for Asian and Hispanic patients compared to Whites. In recent years, significant reduction in mortality was observed for Black, White, and Asian but not Hispanic patients

*CABG* coronary artery bypass grafting surgery, *NIS* National Inpatient Sample, *SES* socioeconomic status, *STS* Society of Thoracic Surgeons, *US* United States, *VHA* Veterans Health Administration

^*^ = This study did not provide aggregated sample size total for all 28 studies

### Mechanisms Underlying Disparities in CABG

Studies seeking to explain the specific etiology of racial/ethnic disparities in survival following CABG have noted that baseline patient characteristics [15], hospital quality [7, 8, 21], health insurance status [22], and SES [15, 22–24] all play a role. Higher prevalence of severe cardiovascular disease prior to CABG is one of the larger contributors of disparities in minority populations [7, 16, 25], as well as additional pre-operative comorbidities [7, 18]. However, these comorbidities in Black patients only explained less than 5% of racial/ethnic disparities in CABG survival outcomes [7]. Konety et al. noted that following adjustment, hospital quality could mitigate mortality differences at 3-months [16], while Rangrass et al. reported hospital quality contributed to 35% of the disparate mortality outcomes observed between minority groups and White patients [7]. Mehta et al. further evaluated the degree to which clinician, hospital, and health care factors account for racial/ethnic disparities in CABG outcomes [8]. In this study, Black patients had higher procedural mortality and morbidity, in part due to patient comorbidities, SES, hospital and surgeon effects (defined with respect to surgery volume and mortality risk ratios), as well as care factors (including use of internal mammary artery and perioperative medication use) [8]. Even after accounting for these differences, Black race remained an independent predictor of worse outcomes [8].

SES has also been considered a risk factor for poor mortality among CABG patients, as it impacts patient insurance status and hospital access [26]. Regardless of race, Medicaid insurance was an independent risk-factor of mortality compared with private insurance and was reported to be associated with worse outcomes after isolated CABG [22]. Coyan et al. also reported that following CABG, patients from lower income quartiles had an increased 5-year mortality [27]. In a recent study, Hannan et al. evaluated the effect of area deprivation index, a granular measure of SES, on short-term CABG mortality. Patients from the most deprived areas had a higher in-hospital deaths following CABG, while Black, Hispanic and Medicaid patients were more likely to be readmitted [28]. In addition, Black patients were more likely to be treated at hospitals with higher risk-adjusted mortality [8]. Together, these studies suggest that poorer outcomes in Black and minority patients are in part due not only to patient factors but also environmental factors such as SES, insurance status and access to care.

## Valvular Surgery

### Surgical Aortic Valve Replacement (SAVR)

Aortic stenosis (AS) is the most common valvular disease world-wide [29], and aortic valve replacement (AVR) is an effective therapy for relieving symptoms and improving survival [30]. While the prevalence of AS in racial and ethnic minority patients is lower than Whites patients [31], following adjustment, surgical intervention, treatment rates and complications are significantly lower in minority patients [32, 33]. Even when afflicted with severe AS, Black patients are still less likely than White patients to undergo any type of AVR (Table 2) [34]. While treatment rates in minority patients are lower, there are mixed survival outcomes. In previous studies, Yeung and colleagues demonstrated that while SAVR was less likely performed in Black patients, 3-year survival was similar [30]. Similarly, McNeely et al. reported that while Black patients had higher 30-day readmission after SAVR, this did not impact mortality [35]. In contrast, Ahmed et al. demonstrated that among Medicare beneficiaries, 1-year mortality was higher for Black patients, while Hispanic, Asian, and American Native patients had lower risk adjusted 1-year mortality [31]. Supporting this finding, Li et al. demonstrated that post-SAVR, Black patients had higher in-hospital mortality [36].

**Table 2:** Racial and ethnic disparities in aortic valve surgery

Author, Year	Study Design	Era	Sample Size	Population and % Ethnic Breakdown	Outcomes
Ahmed et al. [31••]	Retrospective population-based cohort study using Medicare beneficiaries	2010–2018	N = 1,513,455	White (91.3%), Black (4.5%), Hispanic (1.1%), Asian, and North American Native (3.1%)	Adjusted treatment rates for AS was significantly lower for Black, Hispanic, and Asian/North American Native patients compared with Whites. Rate of hospitalization for AS was higher for Black and Hispanic patients compared with Whites. Risk-adjusted 1-year mortality was higher for Black patients, while Hispanic, and Asian and North American Native patients were lower
Taylor et al. [33]	Retrospective study with 3,137 Black and 46, 249 White patients using STS National Cardiac Database who underwent AVR or MVR	1999–2002	**AVR:** N = 34,510 **MVR:** N = 14,876	**AVR:** White (94.9%), Black (5.1%) **MVR:** White (90.8%), Black (9.2%)	Black race was associated with increased risk of postoperative complications after AVR and MVR; but Black race was not a significant predictor of operative mortality after AVR or MVR only
Stamou et al. [90]	Retrospective Study using Massachusetts Cardiac Surgery Database with adults who underwent isolated AVR or AVR with CABG	2002–2008	N = 6,809	White (95.2%), Non-White (4.8%),	No race-based differences in postoperative outcomes between ethnic groups. 30-day mortality rate was 2.8% for Whites, and 3.7% for Non-whites
Yeung et al. [30]	Single-center retrospective and observational cohort study evaluating rates of AVR	2004–2010	N = 880	White (90%), Black (10%)	Black patients underwent AVR less frequently than White patients, had a higher prevalence of comorbidities and refused treatment more often. No differences in 3-year survival outcomes were observed between Black, and White patients who received AVR
Yoon et al. [91]	Prospective cohort study of a multicenter, international Asian TAVR registry examining patients with AS who underwent TAVR in Asian countries	2010–2014	N = 848	Asian only	Following TAVR, Asian patients experienced comparable clinical outcomes compared to previously published trials and observational studies
McNeely et al. [35]	Retrospective cohort study of patients undergoing SAVR using Medicare database	2011–2013	N = 95,078	White (93.9%), Black (4.8%), and Hispanic (1.3%)	Black patients had worse unadjusted 30-day and 1-year mortality than Whites or Hispanics, which did not persist after adjustments. Black patients had higher 30-day readmission rates after SAVR compared to Whites and Hispanics, which persisted after adjustment for comorbidities
Alqahtani et al. [40]	Retrospective study using NIS database who underwent TAVR	2011–2014	N = 7,176	White (95.7%), Black (4.3%)	Black patients had similar utilization rates, in-hospital outcomes, and cost of TAVR between compared to Whites in contemporary practice in the United States
Minha et al. [37]	Single-center, prospective cohort study	2007–2013	N = 469	White (74.5%), Black (10.8%), and Other (15.3%)	TAVR surgery was performed less frequently in Black patients. When performed in Black patients they experienced a higher rate of hemodynamic instability and postoperative intubation but shared similar risks and outcomes compared with Whites
Sleder et al. [10]	Single-center retrospective case–control study of patients with severe AS who underwent TAVR at a major referral center	2013–2014	N = 335	Non-Black (85.1%), Black (14.9%)	Socioeconomic and racial disparities were found in Black patients who underwent TAVR. For every $10,000 increase in income the odds of undergoing TAVR increased by 10%; non-black patients were significantly more likely to undergo TAVR than blacks, with no differences in comorbidities between groups
Alkhouli et al. [39]	Retrospective cohort study of patients undergoing TAVR using STS/American College of Cardiology TVT Registry Database	2011–2016	N = 70,221	White (91.3%), Black (3.8%), Hispanic (3.4%), and Asian/Native American/Pacific Islanders (1.5%)	Racial/ethnic minorities were underrepresented in TAVR. 30-day and 1-year adjusted mortality was similar in Blacks and Hispanics compared with Whites, but lower among Asian/Native American/Pacific Islanders. Black and Hispanic patients had more HF hospitalizations compared with Whites, which persisted after risk-adjustment for SES
Hernandez-Suarez et al. [41]	Retrospective cohort study of patients undergoing TAVR using NIS database	2012–2014	N = 36,270	White (92.0%), Black (4.1%), and Hispanic (3.9%)	Hispanic patients had decreased TAVR utilization, increased in-hospital complications, prolonged length of stay and increased hospital costs; but in-hospital mortality was not associated with race/ethnicity
Roberts et al. [42]	Single-center retrospective chart review of patients who underwent TAVR at a North Dakota community hospital	2012–2021	N = 1153	Non-AI (98.3%), AI (1.7%)	Northern Plains American Indians with severe AS had reduce TAVR utilization, higher rates of DM and smoking history, as well as shorter lifespan following TAVR
Jaiswal et al. [43]	Meta-analysis of three studies (Alkhouli et al., 2019; Minha et al., 2015;Suarez et al., 2019) with TAVR patients	Inception-2022	N = 102,009	White (95.9%), Black (4.1%)	Significant health disparity after TAVR outcomes existed in racial minorities, and Black patients were at higher risk of myocardial infarction and acute kidney injury compared to White patients
Kulkarni et al. [92]	Meta-analysis to determine whether procedure-related complications represent barriers to SAVR and TAVR among Blacks	Inception-2021	**TAVR:** N = 691,963 **SAVR:** N = 1, 144,269	**TAVR:** White (89.6%), Black (4.7%) **SAVR:** White (86.9%), Black (6.4%)	Utilization rate of TAVR and SAVR was significantly less for Black patients compared to Whites. Following TAVR, Blacks experienced more stroke and transient ischemic attack compared to Whites. Survival outcomes was similar between Blacks and Whites for both SAVR/TAVR
Brennan et al. [34]	Retrospective cohort study with symptomatic severe aortic valve stenosis were identified via Optum’s deidentified electronic health records database	2007–2017	N = 32 853	White (96.2%), Black (3.8%)	Black individuals were less likely than Whites to receive AVR, but differences have been narrowed
Li et al. [36]	Retrospective cohort study of patients who underwent SAVR and TAVR for aortic stenosis were identified in NIS	2015–2020	**TAVR:** N = 53,827 **SAVR:** N = 53,645	**TAVR:** White (84.41%), Black (4.0%) **SAVR:** White (78.52%), Black (5.59%)	Black patients were underrepresented in both SAVR and TAVR, and had longer wait times to surgery, longer hospital stays and higher hospital charges. Following surgery, Blacks experienced higher in-hospital mortality following SAVR, but comparable outcomes were observed in TAVR compared to Whites

*AI* American Indians, *AS* aortic stenosis, *AVR* aortic valve surgery, *DM* diabetes, *HF* heart failure, *LOS* length of stay, *NIS* National Inpatient Sample, *SES* socioeconomic status, *SAVR* surgical aortic valve replacement/repair, *STS* Society of Thoracic Surgeons, *TAVR* transcatheter aortic valve replacement, *TVT* Transcatheter Valve Therapy

### Minimally Invasive Aortic Valve Surgery

The development of transcatheter aortic valve replacement (TAVR) has increased treatment options for patients with AS, and the number of aortic valvular procedures performed has drastically increased over the last decade. Several studies have demonstrated that racial and ethnic minority patients are underrepresented in TAVR procedures [10, 36, 37], with higher utilization and lower mortality rates among White recipients compared to racial/ethnic minority groups [38]. In spite of this, following TAVR, Black patients have similar 30-day and 1-year survival, and clinical outcomes [37, 39]. In contrast, Alqahtani et al. reported that there was no significant difference in utilization among White and Black patients [40], while McNeely et al. demonstrated that likelihood of readmission or discharge to home after TAVR were not associated with race [35]. Conversely, despite TAVR underutilization, Hispanic patients had higher in-hospital complications, prolonged length of stay, and increased hospital costs compared with Black and White patients [41]. Americans Indians also had reduced survival post TAVR [42]. Jaiswal et al. also demonstrated that following TAVR, Black patients are at higher risk of myocardial infarction and acute kidney injury [43].

There are only limited studies investigating racial/ethnic disparities in TAVR/SAVR outcomes among Asian and Native Americans. Li et al. reported that, like Black patients, Asian Americans were underrepresented in AVR, and despite having similar post-TAVR outcomes to Whites, Asian Americans faced greater risks of post-operative SAVR mortality and surgical complications [44]. In the only study to evaluate disparities in TAVR/SAVR among Native Americans, Li et al. reported that these individuals were more likely to undergo SAVR than TAVR. Following propensity matching, however, Native Americans had five times higher stroke and three times higher venous thromboembolic events after SAVR [45].

Despite conflicting mortality results, these data demonstrate that Black patients had higher peri- and post-operative complications, which requires further interventions to address. As these studies focused on in-hospital or acute short-term mortality, further studies are needed to assess long-term mortality (> 5-years), and the underlying causes of the disparity in TAVR use. In addition, the limited data on Asian and Native Americans suggested a critical need for further studies to elucidate the progression of racial inequities and targeted actions to deliver equitable care [44, 45].

### Mechanisms Underlying Disparities in SAVR/TAVR

Racial/ethnic disparities in SAVR/TAVR are impacted largely by health insurance status, SES, and patient factors [46, 47]. In a cross-sectional study of Medicare beneficiaries that investigated whether TAVR/SAVR procedures are preferentially available to Whites patients, Gupta and colleagues reported that AVR within 6-months of AS admission is lower for Black, Hispanic, and Asian patients [48]. In metropolitan US TAVR programs, following adjustments for age and comorbidities, zip codes with higher density of Black and Hispanic patients and individuals with greater SES disadvantages had lower utilization of TAVR [49]. However, it is unclear whether these data reflect reduced incidence of symptomatic AS or disparate use of TAVR by minority groups, and therefore requires further study. In contrast, following adjustments for SES, Alkhouli et al. noted that 1-year adjusted mortality was similar among Black and Hispanic patients following TAVR, but lower among patients of Asian/Native American/Pacific Islander race [39]. Together, these data suggest racial/ethnic disparities in access and post-operative management contribute to racial/ethnic disparities in patients undergoing TAVR/SAVR. However, further studies are required to elucidate specific causes racial/ethnic disparities in TAVR/SAVR use.

### Mitral Valve Surgery

Analogous to aortic surgery, White patients have a higher utilization of mitral valve surgery (MVS) or repair compared with Black patients (Table 3) [39, 50]. Early studies demonstrated that Black patients undergoing MVS were younger and had more comorbid conditions including diabetes mellitus, renal failure, congestive heart failure, endocarditis, and rheumatic disease [51]. There were no differences, however, in postoperative complications and hospital mortality. In a subsequent study, Vassileva et al. also reported that racial and ethnic minority patients had a higher incidence of comorbidities, which resulted in prolonged hospitalization following MVS; however, no race-based differences in short-term mortality were noted [52]. In a multicenter study, following adjustments for pre-operative factors, mitral etiology, and hospital quality, race was not associated with MVS complications or mortality. However, Black patients had increased utilization of extended care facilities and rates of readmission [53].

**Table 3:** Racial and ethnic disparities in mitral valve surgery

Author, Year	Study Design	Era	Sample Size	Population and % Ethnic Breakdown	Outcomes
DiGiorgi et al. [51]	Single-center retrospective cohort study of patients with isolated mitral valvuloplasty or mitral valve replacement	1993–2003	N = 1,425	White (91.4%), Black (8.6%)	Black patients were younger with higher pre-operative comorbid risk factors including diabetes mellitus, renal failure, congestive heart failure, endocarditis, and rheumatic mitral disease. No significant differences in postoperative complications or hospital mortality
Vassileva et al. [52]	Retrospective cohort study of patients who underwent MV repair or replacement using NIS database	2005–2007	N = 35,074	Whites (77.7%), Black (8.4%), Hispanics (7.0%), and Others (6.9%)	Non-white patients were more likely to be younger, on Medicaid, from urban locations and present in urgent/emergent basis. Black and Hispanic patients were less affluent with higher Charlson comorbidity index compared to Whites. No differences in-hospital mortality, but increased LOS was observed in Blacks and Hispanics
Pienta et al. [53]	Retrospective cohort study of patients with mitral valve repair or replacement with or without CABG in the state of Michigan	2011–2020	N = 9074	White (86.6%), Black (11.1%), and Other. (2.3%)	Preoperative STS risk score was higher in Black patients compared to White and Others, due to higher incidence of diabetes, HTN, and chronic lung disease. While operative mortality was similar, Black patients had higher odds of extended care facility utilization and readmission
Malas et al. [58]	Multi-center retrospective cohort study of Medicare and Medicaid beneficiaries undergoing isolated first-time mitral repair	2012–2019	N = 10,322	DCI < 80 (90.3%), DCI ≥ 80 (9.7%)	Patients from distressed communities underwent surgery at lower volume centers and traveled further for surgical care. Community distress was independently associated with 3-year mortality and heart failure readmissions
Shechter et al. [55]	Single-center, retrospective analysis of consecutive TEER procedures	2013–2020	N = 964	White (77.9%), Black (9.1%), Asian (7.1%), and Hispanic (5.9%)	Non-whites and Blacks were younger and more likely be female, from lower socioeconomic areas and were not fully insured. Among patients with functional MR, Non-whites (vs. whites) and blacks (vs. Non-blacks or whites) experienced higher in-hospital mortality at 1-year post TEER
Sparrow et al. [11]	Retrospective cohort-based observational study of patients undergoing TEER using the NIS	2013–2018	N = 3795	White (86.2%), Black (7.4%), and Hispanic (6.4%)	Black and Hispanic patients were underrepresented in TEER procedures. While Black patients had higher rates of deaths, no differences in in-hospital complications were observed between groups
Steitieh et al. [57]	Retrospective multistate analysis using HCUP’s SID for 2016 TEER hospitalizations across several states	2016	N = 1567	Whites (83.8%), Blacks (5.9%), Hispanics (5.3%), and Other (5.0%)	Black and Hispanic patients were less likely to undergo TEER at high-volume centers and had more comorbidities compared with White patients. Hispanic patients were 3 times more likely to die during indexed TEER admission compared to Whites
Glance et al. [50•]	Cross-sectional study using data from the STS Database for patients who underwent mitral valve surgery	2014–2019	N = 103 753	White (85.8%), Black (10.0%), and Hispanic (4.2%)	Following adjustment, Black patients were less likely to undergo MIMVS, had higher in-hospital mortality, and experienced a major complication compared to White patients. Black patients undergoing MIMVS were more likely to have Medicaid insurance or receive care from low-volume surgeons
Spring et al. [59]	Retrospective study of patients undergoing TEER using the NIS	2016–2017	N = 10,195	Urgent TEER (24.2%), Non-urgent TEER (75.8%) Whites, Blacks, and Hispanics*	Hispanic race, Medicaid insurance, and low-income status were associated with increased likelihood of urgent hospital admission and TEER. Urgent TEER was associated with increased mortality, prolonged LOS, and increased cost
Ismayl et al. [54]	Retrospective cohort study of hospitalizations for transcatheter mitral valve replacement using NIS database	2016–2020	N = 5005	White (76.7%), Black (10.1%), Hispanic (6.3%), and Other (6.9%)	No significant differences in in-hospital mortality and procedural complications, even after adjustment for baseline characteristics. Following adjustments, vascular complications were higher in Blacks compared to White patients

*CABG* coronary artery bypass grafting surgery, *DCI* Distressed Community Index, *HTN* hypertension, *HCUP* Healthcare Cost and Utilization Project, *LOS* length of stay, *NIS* National Inpatient Sample, *MIMVS* minimally invasive mitral valve surgery, *MR* mitral regurgitation, *MV* mitral valve, *SES* socioeconomic status, *SID* State Inpatient Database, *STS* Society of Thoracic Surgeons, *TEER* transcatheter edge to edge repair, *US* United States

^*^ This study evaluated race in multivariate analysis only

Race-based survival outcomes have also been reported among patients undergoing minimal invasive mitral valve surgery (MIMVS). In one study, Black and Hispanic patients undergoing transcatheter mitral valve repair had similar in‐hospital outcomes compared with White patients, except for higher incidence of vascular injury among Black patients [54]. In a recent cross-sectional study, Glance et al. noted that Black individuals were less likely to undergo MIMVS, even after adjusting for patient risk, and had higher risk of in-hospital mortality and major complications [50]. Black, White, Hispanic, and Asian patients who underwent transcatheter Edge-to-Edge Repair (TEER) for mitral regurgitation exhibit major differences in baseline characteristics and, intra-procedurally more devices were implanted in Black patients suggesting more advanced disease [55]. At 1-year, both minority and Black patients experienced lower survival and increased heart failure hospitalizations [47]. In an additional study, Black and Hispanic patients were less likely to undergo TEER, and Hispanic patients were three times more likely to experience in-hospital mortality after TEER than White patients [56]. Supporting this finding, Sparrow and colleagues demonstrated that Black patients experienced a higher risk of in-hospital death, but similar overall incidence of post-procedural adverse events [11].

### Mechanisms Underlying Disparities in Mitral Valve Surgery

Some of the potential processes driving these variations in clinical outcomes may be connected to hospital quality, SES, and insurance status. For example, Glance et al. reported that the infrequency of Black patients undergoing MIMVS was in part due to insurance status [50]. Patients with commercial insurance had greater than two-fold higher odds of undergoing MIMVS than individuals with Medicaid insurance. Moreover, Black patients were more likely to have Medicaid insurance and undergo MIMVS at Low volume (LV) centers (< 20 cases) [50]. Similarly, Steitieh et al. reported that racial/ethnic minorities, particularly Black and Hispanic patients, are less likely to undergo TEER at High volume (HV) centers [57]. Interestingly, the authors also demonstrated geographic clustering of TEER centers, with a higher ratio of White patients in zip codes with HV TEER centers compared with LV TEER centers, which had a higher density of minorities [57].

Differences in income and insurance status also impact survival outcomes among patients undergoing MVS (Table 3) [11, 55, 58]. In early studies, Vassileva and colleagues demonstrated that Black and Hispanic patients undergoing MVS tended to be less affluent [44]. Malas and colleagues investigated the influence of SES on survival after mitral repair among Medicare beneficiaries independent of race and ethnicity [58]. Patients from distressed communities, which incorporates education level, poverty, unemployment, and housing security, were more likely to undergo surgery at LV centers and traveled further for surgical care. At 3-years, unadjusted survival and cumulative incidence of heart failure readmission were worse in patients from distressed communities. Community distress was independently associated with 3-year mortality and heart failure readmissions [58]. This finding is supported by Shechter et al. who demonstrated that racial/ethnic minorities undergoing TEER were from lower SES areas, not fully insured, more often diagnosed with functional mitral regurgitation, and more often affected by biventricular dysfunction [55]. Sparrow et al. also showed that patients with from lower SES (income quartile-1) had worse in-hospital outcomes, with increased cardiac and vascular events, compared with higher SES (quartile-4) [11]. Unequal access to monitoring and preventive care based on race and SES is best exemplified by the higher use of urgent TEER among Black patients [59]. Using NIS database, Spring and colleagues noted that Hispanic race, Medicaid insurance and patients with low incomes undergoing TEER have increased morbidity and mortality, prolonged length of stay, and increased hospital cost [59]. As such, increasing access to private insurance and HV centers could improve minority patient outcomes in cardiac surgery.

## Cardiac Transplantation

Several hundred-thousand people in the United States are currently living with end-stage heart failure for which transplantation is the only viable treatment option. However, only roughly 1% of these people end up being transplanted [60]. This low percentage of transplantation utilization is due to several factors, including the lack of suitable donors. As such, this scare resource is only afforded to patients who qualify based on stringent guidelines, which are both subjective and objective [61]. The subjective evaluation is mainly based on psychosocial requirements, suitable care, and support groups. Unfortunately, this subjectivity leaves much up for interpretation and unequitable decision making.

Though heart transplantation has drastically increased in use since its inception [6] and has improved in terms of patient outcomes and decreasing waitlist times – there are still challenges relative to making this treatment option equitable. Since 1987, there have been over 75,000 heart transplants in the United States, of which the vast majority have been White recipients (72%) [62]. Over this time, Black recipients comprised 7% of total recipients in 1987, which has increased to 26% of total recipients in 2019 [62]. Additionally, Hispanic patients make up ~ 8% of recipients [63]. While Black recipients are becoming more represented within the transplant population, Hispanic patients are still under-represented. Heart transplantation inequities and disparate outcomes still persist, however, for Black recipients. A recent study by Cogswell et al. noted that Black patients have markedly lower rates of transplantation and have correspondingly increased waitlist mortality [64]. Even in centers that serve racially and ethnically diverse populations, Black and Hispanic patients have a lower likelihood of receiving a transplant versus White patients [65].

Regarding mortality, a comprehensive UNOS analysis noted that White patients had much higher survival over the first several decades of transplantation (1987–2016). This mortality difference has subsequently subsided in recent years (2017–2020) [62]. Moayedi et al. reported, however, that from 2013–2017 Black recipients experienced significantly higher mortality versus White recipients based on the Outcomes AlloMap Registry [1]. Similarly, Chouairi et al. noted that Black and Hispanic recipients have an increased mortality following transplantation over a more recent time period (2011–2020), even after adjusting for the new allocation system that was implemented in 2018 [66]. While the data are conflicting, more recent evidence supports that minority races have worse survival after heart transplantation [1, 66].

While large database studies are important to study post-operative mortality, their lack of granular data makes it difficult to discern the reasons for these disparities amongst races. There is evidence to suggest that focusing on unique immunologic contributors to racial/ethnic disparities can help reduce graft failure and improve outcomes in heart transplantation [67]. Prior studies demonstrate that immunologic factors confer the greatest risk for incident graft failure and is highest in Black population [68]. Therefore, strategies to improve induction and maintenance of immunosuppression Black patients have been prioritized [2, 15]. Black and Hispanic recipients more often have public insurance and have end-stage renal disease at the time of transplant. Additionally, minority patients spend significantly longer on the waitlist [66]. In general, Black patients also have higher risk of developing and succumbing to heart failure. Furthermore, Black patients are less likely to be under the care of a cardiologist, which may also contribute to these inequities [69]. Another layer to this issue is the complex interplay of race and SES. Wayda et al. noted that recipients experiencing socioeconomic disadvantage and Black recipients had lower survival. Importantly, individuals living in the most disadvantaged areas had almost the exact same hazard ratio for mortality as Black patients [70]. Recently, Azap et al. reported that high social vulnerability index (SVI) was independently associated with increased mortality following heart transplantation even if recipients survived at least one year [71] (Table 4).

**Table 4:** Racial and ethnic disparities in cardiac transplantation

Author, Year	Study Design	Era	Sample Size	Population and % Ethnic Breakdown	Outcomes
Moayedi et al. [1]	Retrospective review of AlloMap database	2013–2017	N = 933	White (79%), Black (21%)	Black patients had an increased adjusted mortality risk. Higher tacrolimus levels were associated with decreased mortality in Black patients. FLT3 upregulation was associated with increased mortality in Black patients
Trivedi et al. [62]	Retrospective review of UNOS database	1987–2020	N = 67,824	White (71.9%), Black (16.6%)	Proportion of Black patients receiving transplants increased from 5% in 1987 to 26% in 2019. Survival of Black patients gradually increased
Blitzer et al. [63]	Retrospective review of UNOS database	2000–2018	N = 41,841	White (71%), Black (20%), Hispanic (8%)	Black and Hispanic patients had decreased survival compared to White recipients following transplant. Racial differences existed in donor and recipient characteristics, and recipient outcomes after transplantation
Cogswell et al. [64]	Retrospective review of UNOS database	2015–2021	N = 17,384	White (72%), Black (28%)	The rate of transplantation, and rate of delisting worsened for Black compared to White patients with the introduction of the new allocation system in 2018
Chouairi et al. [66]	Retrospective review of UNOS database	2011–2020	N = 32,353	White (66%), Black (25%), Hispanic (9%)	Although there was an increase of Black and Hispanic patients listed for transplantation, Black patients are still less likely to be transplanted (despite allocation change in 2018) and had a higher risk of post‐transplantation mortality
Morris et al. [68]	Retrospective review of UNOS database	2004–2012	N = 15,255	White (75%), Black (17%), Hispanic (7%)	Black and Hispanic patients are more likely to have risk factors for graft failure: low education, public insurance, allosensitization, higher human leukocyte antigen mismatch, non-adherence, and history of rejection requiring hospitalization
Breathett et al. [69]	Retrospective review of Premier database	2010–2014	N = 104,835	White (80.3%), Black (19.7%)	Black Patients were less likely than White Patients to receive care by a cardiologist. Care by a cardiologist was associated with higher survival for both races
Breathett et al. [65]	Retrospective review of UNOS database	2016–2018	N = 5,502	White, Black, Hispanic ^**#**^	Black patients in Nevada, Missouri, Georgia, and New York had a higher-than-expected mortality following transplantation compared to White patients
Wayda et al. [70]	Retrospective review of UNOS database	1994–2014	N = 33,893	White (74%), Black (16%)	Racial and socioeconomic disparities existed following transplantation, but this difference may be narrowing over time
Azap et al. [71]	Retrospective review of UNOS database	2006–2020	N = 27,740	White (65.8%), Black (21.7%)	High or average SVI was independently associated with increased mortality following transplantation in patients with 1-year conditional survival
Breathett et al. [6]	Retrospective review of UNOS database	2018–2023	N = 159, 177	White (69.1%), Black (30.9%)	Cumulative incidence of heart offer acceptance was lower for Black candidates compared to Whites Disparities persisted after adjusting for several variables

*FLT3* fms like tyrosine kinase 3, *UNOS* United Network for Organ Sharing, *SVI* social vulnerability index, ^**#**^ % break down not available

The solution to eliminate these disparities are multifaceted and must tackle several social constructs [72, 73]. Though evaluating psychosocial factors is necessary for all transplant patients, it can add a certain amount of explicit or implicit bias to determine who is suitable for transplant. Breathett et al. conducted a national survey study of heart failure physicians (N = 422) and noted that even with identical clinical and psychosocial attributes, heart transplantation was less likely to be recommended to the Black patients due to concerns about trustworthiness and psychosocial factors [74]. Thus, it is necessary to further study and carefully examine the process in which we determine whether or not someone is psychosocially fit for heart transplantation. Ultimately, transplant providers must make every effort to understand the causes behind disparities and issues surrounding the equitable use of heart allografts, as this is the best way to implement change. Previous studies have highlighted the importance of improving minority involvement within clinical trials [2], the number of diverse health care professionals within the medical field and finally improving social policies that may drive health disparities [66, 67, 75].

### Durable Mechanical Circulatory Support

While the gold standard of treatment for end-stage heart failure is eventual transplantation, sometimes that is not feasible. With improvingly durable mechanical circulatory support (MCS), more patients who do not qualify for transplant are turning to MCS options. While Black adults currently make up around 12.1% of US population, these individuals currently represent around 29.5% of the adult heart failure population, and 27.5% of left ventricular device implantations (LVAD) [76]. Though Black patients made up similar proportions of LVAD recipients compared with their prevalence of heart failure, these patients still received LVADs at a significantly lower rate versus White patients [76]. Studies by Cascino et al. and others also noted a similar reduced utilization of all ventricular assist devices (VAD) among Black patients [77, 78]. In conclusion, similar to heart transplantation, providers must strive to eliminate bias, evolve psychosocial testing, and continue to study disparity related gaps in healthcare to make this therapy equitable for all (Table 5).

**Table 5:** Racial and ethnic disparities in mechanical circulatory support

Author, Year	Study Design	Era	Sample Size	Population and % Ethnic Breakdown	Outcomes
Rose et al. [76]	Retrospective review of patient discharge records from 20 states	2010–2018	N = 1,894,878	White (59.8%), Black (20.1%), and Hispanic (12.7%)	Among adults with heart failure with reduced ejection failures, the use of left-ventricular assist device and transplantation was lower among women and Black men
Cascino et al. [77]	Observational cohort study of registry evaluation of vital Information for VADs in Ambulatory Life	2015–2016	N = 377	White (73.5%), Black (26.5%)	For patients receiving care by advanced heart failure cardiologists at VAD centers, there was less utilization of VAD and transplant for Black patients even after adjusting for severity, quality of life, and social determinants of health
Cascino et al. [78]	Retrospective review of Medicare patients	2008–2014	N = 12,310	White (77.1%), Black (22.9%)	Disparities in left-ventricular assist device use by race and sex existed and were not explained by clinical characteristics or social determinants of health. The treatment and post- left-ventricular assist device survival by race were equivalent

*VAD* ventricular assist device, *LVAD* left ventricular assist device

## Future Directions and Strategies to Improve Cardiac Surgery Outcomes in Racial/Ethnic Minority Groups

With an increasingly diverse US population, cardiac surgeons must recognize and address drivers of racial/ethnic disparities in cardiac surgery (Fig. 1). Efforts should begin with addressing racial/ethnic minority representation in study populations and clinical trials [79, 80]. Recent assessment of racial minority representation in cardiac surgery randomized clinical trials (RCTs) by Cancelli and colleagues demonstrated that only 9 of the 51 RCTs published between 2000 and 2020 reported the race of enrolled participants [80]. When reported, only 11.2% were non-White, despite racial/ethnic minorities comprising over 40% of US population. In a separate study, using the national clinical trials database, of the 178 cardiovascular trials identified between 2008 and 2017, only 72 (42.7%) and 52 (29.2%) reported racial/ethnic (Hispanic vs non-Hispanic) distribution, respectively [81]. Furthermore, Black (4%) and Hispanic (11%) patients were underrepresented, while Asian Americans (10%) were overrepresented. In addition to historical patient mistrust, rigid research design with restricted eligibility criteria, language, and education requirements, as well as limited outreach continue to perpetuate this disparity [2, 77]. Initiation of prospective registry-based studies [46] and prioritized recruitment of minorities [82, 83] with culturally sensitive government mandates may improve understanding of specific causes of the race-based differences in outcomes.

Racial/ethnic minority groups also receive more urgent cardiac surgeries at LV facilities with higher risk of morbidity and death [59]. Systematic employment of multidisciplinary teams involving cardiology, cardiac surgery, anesthesiology, critical care, medical ethics, and health disparity experts in safety net LV hospitals is another avenue to improve access and increase early referrals for minority patients. The greater ratio of LV centers to HV centers in disadvantaged regions provide historical evidence for their increased use by racial/ethnic minorities. Therefore, State, and Federal funding policies focusing on these LV safety net hospitals offers a chance for reform and better care for minority communities [84]. In some reports, Black patients refused surgical interventions more often than Whites for certain procedures [30, 85], which may highlight historic mistrust for the medical enterprise. Reduced offering of transplantation to the Black patients further highlights clinical implicit biases [74]. The high level of Black and Hispanic readmissions for several cardiac surgery procedures also suggests disparate access to post-operative surveillance and preventive care [29, 53, 58]. Increasing availability of cardiac rehabilitation therapy [12, 13] for racial/ethnic minorities especially those from low SES is essential to reducing disparate outcomes. Improving diversity of cardiac surgeons is also necessary for attenuating readmission and increase favorable patient outcomes. Research has demonstrated that racially diverse medical teams can improve multicultural care, which ultimately improves patient satisfaction [86]. Given the perceived benefits of doctor-patient race concordance and culturally sensitive interactions on physician–patient communication, the fact that less than 3% of US cardiac surgeons are Black/African American, and 4% Hispanic may also impact racial/ethnic disparities [87]. Towards equitable representation, coordinated efforts should be made to improve the mentoring pipeline and development of young aspiring cardiac and cardiothoracic surgeons from Black and Hispanic groups [87, 88], which some organizations have done by creating scholarships and grants designed specifically for underrepresented minorities [89].

## Limitations

There are limitations to this review. Though our literature review was broad and based on expert knowledge within this field of study, we undoubtably excluded studies that perhaps met inclusion criteria. In addition, due to the complex relationship between race and cardiac surgery outcomes, even in large multicenter studies, it is difficult to elucidate specific underlying causes of racial/ethnic disparities. Moreover, studies using populations with high-risk features may confound the effects of race, making it more difficult to assess the independent risks related to race [1].

## Conclusions

The underlying etiology of racial/ethnic disparities in cardiac surgery is partly related to access to healthcare, lower incidence of utilization, disparate differences in income, zip code, surgeon bias, and hospital quality. Notwithstanding, intersectional considerations are limited. Therefore, large-scale national programs are necessary to dissect specific contributors. We recommend using a health equity lens to create an intervention framework that takes into consideration the impact of race/ethnicity, and interrelated variables towards reducing race-based mortality burden and improving care for all patients.

## Key References

- Becker ER, Granzotti AM (2019) Trends in In-hospital Coronary Artery Bypass Surgery Mortality by Gender and Race/Ethnicity --1998-2015: Why Do the Differences Remain? Journal of the National Medical Association 111:527–539.
  - Becker et al. demonstrated that racial disparities in CABG surgery still exist despite overall progress in reducing CABG surgery mortality across all groups in a national sample with over 5 million patients. Specifically, Black males had 35.1% higher adjusted in-hospital mortality following CABG, while Hispanic, and Asian-Americans had significantly lower mortality rates (-9.7% and -17.9% respectively) compared to White patients.

- Rangrass G, Ghaferi AA, Dimick JB (2014) Explaining Racial Disparities in Outcomes After Cardiac Surgery: The Role of Hospital Quality. JAMA Surg 149:223.
  - Rangrass et al. delineated the impact of SES, hospital quality and patient factors on racial disparities in cardiac surgery. Among Medicare enrollees between 2007-2008, risk-adjusted in-hospital mortality for non-white patients was 33% higher than White patients following CABG. However, hospital quality explained only 35% of the observed disparity, while 53% is explained by SES and hospital quality combined.

- Mehta RH, Shahian DM, Sheng S, O’Brien SM, Edwards FH, Jacobs JP, Peterson ED (2016) Association of Hospital and Physician Characteristics and Care Processes With Racial Disparities in Procedural Outcomes Among Contemporary Patients Undergoing Coronary Artery Bypass Grafting Surgery. Circulation 133:124–130.
  - Mehta et al. demonstrated that mortality and major morbidity rates after CABG are higher in Blacks than in Whites. Furthermore, Black race remained an independent predictor of outcomes even after adjusting for surgeon, hospital, and care processes in addition to patient and SES factors.

- Ahmed Y, Van Bakel PAJ, Hou H, et al (2023) Racial and ethnic disparities in diagnosis, management and outcomes of aortic stenosis in the Medicare population. PLoS ONE 18:e028181.
  - Ahmed et al. demonstrated disparate cardiac surgery utilization and outcomes among patients with aortic stenosis in a population-based cohort over 1.5 million Medicare beneficiaries between 2010-2018. Black, Hispanic, and Asian/North American Native patients had significantly lower adjusted treatment rates for aortic stenosis compared with Whites. Despite higher rates of hospitalization due to aortic stenosis among Black and Hispanic patients, the risk-adjusted 1-year mortality was higher for Black patients, while Hispanic, and Asian and North American Native patients were lower compared to Whites. The authors delineate the enduring racial and ethnic disparities in acute and long-term outcomes following aortic surgery.

- Glance LG, Joynt Maddox KE, Mazzefi M, et al (2022) Racial and Ethnic Disparities in Access to Minimally Invasive Mitral Valve Surgery. JAMA Netw Open 5:e2247968.
  - Glance et al. demonstrated that income and insurance type may contribute to racial/ethnic disparities in minimally invasive mitral valve surgery (MIMVS). While Black patients are less likely to undergo MIMVS, adjusted in-hospital mortality and incidence of a major complication was higher in Black patients compared to White patients. Furthermore, Black patients undergoing MIMVS are more likely to have Medicaid insurance and receive care from low-volume surgeons.

## Data Availability

No datasets were generated or analysed during the current study.

## Abbreviations

AS: Aortic stenosis
AVR: Aortic valve replacement
CABG: Coronary artery bypass graft
HV: High volume
LV: Low volume
LVAD: Left ventricular assisted device
MCS: Mechanical circulatory support
MIMVS: Minimally invasive mitral valve surgery
SAVR: Surgical aortic valve replacement
SES: Socioeconomic status
STS: Society of Thoracic Surgeons
TAVR: Transcatheter aortic valve replacement
TEER: Transcatheter edge to edge repair
VAD: Ventricular assisted device
